# 

**Published:** 1937

**Authors:** 


### The Medical Library of the University of Bristol

WITH WHICH IS INCORPORATED

**The Library of the Bristol Medico-Chirurgical Society.**

---

*The following donations have been received since the publication of the list in October,* 1937.

December, 1937.

| | |
|---|---|
| The Hunterian Society (1) .. .. .. | 1 volume. |
| Army Medical Library, Washington, U.S.A. (2) | 2 volumes. |
| Professor E. W. Hey Groves (3) .. .. | 14 ,, |
| Dr. F. S. Hunter (4) .. .. .. .. | 1 volume. |
| Mrs. Lilian Lindsay (5) .. .. .. | 1 ,, |
| Michell Clarke Fund (6) .. .. .. | 1 ,, |
| Professor J. A. Nixon (7) .. .. .. | 2 volumes. |
| Royal College of Surgeons (8) .. .. | 1 volume. |

Unbound periodicals have also been received from Professor E. W. Hey Groves, Professor C. Bruce Perry, Dr. J. O. Symes.

### THE ONE HUNDRED AND SIXTY-FIRST LIST OF BOOKS.

The figures in round brackets refer to the figures after the names of the donors. The books to which no such figures have been attached have either been bought from the Library Fund or received through the *Journal.*

| | | |
|---|---|---|
| *Army Medical Library, Washington, D.C.* | *One Hundredth Anniversary,* 1936.. .. .. .. .. .. .. .. .. (2) | 1937 |
| **Bailey, Hamilton** .. | *Physical Signs in Clinical Surgery* 6th Ed. | 1937 |
| **Bernard, Claude** .. | *Introduccio a l'estudi de la Medicina Experimentale* .. .. .. .. .. .. .. .. (7) | 1937 |
| **Browning, Ethel** .. | *Toxicity of Industrial Organic Solvents. (Industrial Health Research Board Report No.* 80) .. .. .. .. .. .. .. .. (7) | 1937 |
| **Clay, Henry H...** .. | *The Sanitary Inspector's Handbook* 3rd Ed. | 1937 |
| **Colyer, Sir Frank** .. | *Variations and Diseases of the Teeth of Animals* .. .. .. .. .. .. .. (5) | 1936 |

| | | | |
|---|---|---|---|
| **Cox, Gladys M.** .. | *Clinical Contraception* .. .. .. | 2nd Ed. | 1937 |
| **Crawford, A. Muir** .. | *Materia Medica for Nurses* .. .. | 4th Ed. | 1937 |
| **Cruickshank, D. B.** .. | *Tuberculosis, Cancer and Zinc* .. .. | (4) | 1937 |
| **Duke-Elder, Sir W. Stewart** | *Textbook of Ophthalmology* .. | Vol. II (6) | 1938 |
| **East, Terence** .. .. | *Failure of the Heart and Circulation* .. .. | | 1937 |
| **Gabriel, W. B.** .. .. | *Principles and Practice of Rectal Surgery* | 2nd Ed. | 1937 |
| **Glaister, J., and Brash, J. C.** | *Medico-Legal Aspects of the Ruxton Case* | | 1937 |
| **Gould, Sir A. P.** .. | *Elements of Surgical Diagnosis* | 8th Ed. (3) | 1937 |
| **Griffin, F. W. W.** .. | *Scientific Basis of Physical Education* .. .. | | 1937 |
| **Haldin-Davis, H.** .. | *Skin Diseases in General Practice* | 3rd Ed. | 1937 |
| **Hewer, Evelyn E.** .. | *Textbook of Histology for Medical Students* | (3) | 1937 |
| *Index Catalogue of the Surgeon General's Office*, 4th Series, Vol. II. | | (2) | 1937 |
| **Kappis (Max)** .. .. | *Allgemeine und spezielle chirurgische Diagnostik* | (3) | 1937 |
| *League of Nations.* | *Final Report of the Mixed Committee on the Relation of Nutrition to Health, Agriculture and Economic Policy* .. .. .. .. .. .. .. | | 1937 |
| **Lewis, Sir Thomas** .. | *Diseases of the Heart* .. .. .. | 2nd Ed. | 1937 |
| **Luhmann, Kurt** .. | *Die Technik der Operation der Gaumenspalten und Gaumenlippenspalten* .. .. .. | (3) | 1937 |
| **McLaggan, J. Douglas** | *Diseases of the Ear, Nose and Throat* .. .. | | 1937 |
| **Maingot, Rodney** (ed.) | *Post-graduate Surgery.* Vol. III. .. .. | (3) | 1937 |
| **Ombredanne, J., and Mathieu, P.** | *Traité de chirurgie orthopédique* 5 Vols. .. .. .. .. .. .. .. .. .. | (3) | 1937 |
| **Price, Frederick** (ed.) | *Textbook of the Practice of Medicine* (Also India paper copy.) | 5th Ed. | 1937 |
| **Rose and Carless** .. | *Manual of Surgery.* 15th Ed. by Wakeley and Hunter, 2 Vols. .. .. .. .. .. .. | (3) | 1937 |
| **Shands, A. R.** .. .. | *Handbook of Orthopædic Surgery* .. .. | (3) | 1937 |
| **Souttar, H. S.** .. .. | *The Art of Surgery* .. .. .. | 3rd Ed. (3) | 1937 |
| **Steen, Robert Elsworth** | *Infants in Health and Sickness* .. .. .. | | 1937 |
| **Todd, A. T.** .. .. | *Treatment of some Chronic and 'Incurable' Diseases* .. .. .. .. .. .. .. .. .. .. | | 1937 |
| *Treatment in General Practice.* Vol. I. .. .. .. .. .. .. .. .. | | | 1937 |
| **Voronoff, Serge** .. | *Results followed up for twenty years after grafting of the Thyroid, in cases of Myxedematous Cretinism* .. .. .. .. .. .. .. | | 1937 |
| **Williams, Leonard** .. | *Minor Maladies* .. .. .. .. | 7th Ed. | 1937 |
| **Wilton, Ake** .. .. | *Tissue Reactions in Bone and Dentine* .. | (3) | 1937 |

### TRANSACTIONS, REPORTS, JOURNALS, ETC.

| | | |
|---|---|---|
| Calendar of the Royal College of Surgeons, 1937 .. .. .. | (8) | 1937 |
| The Medical Directory, 1938 .. .. .. .. .. .. .. .. .. .. .. .. | | 1938 |
| Quarterly Cumulative Index Medicus. Vol. 21 .. .. .. .. .. .. | | 1937 |
| Saint Bartholomew's Hospital Reports. Vol. LXX. .. .. .. .. | | 1937 |
| Smithsonian Institution Annual Report, 1936 .. .. .. .. .. .. | | 1937 |
| Transactions of the Hunterian Society. Vol. I. 1936–37 .. | (1) | 1937 |
| Transactions of the Ophthalmological Society of the United Kingdom. Vol. LVII, Part I. .. .. .. .. .. .. .. .. .. .. .. .. .. | | 1937 |

### Publications Received

From THE TRUSTEES OF THE BRITISH MUSEUM :

*The Bed Bug.* (British Museum (Natural History) Economic Series No. 5.) By A. W. McKENNY HUGHES, D.I.C. Price 6d.

From HANOVIA LTD., BATH ROAD, CIPPENHAM, SLOUGH :

*Pre-eminent in the Relief of Pain.*

From HOGARTH PRESS :

*Diet and High Blood Pressure.* By I. HARRIS. Price 10s. 6d.

From H. K. LEWIS & CO. LTD. :

*The Sanitary Inspector's Handbook.* By HENRY H. CLAY, F.R.San.I., F.I.S.E. 3rd Edition. Price 16s. 6d.

*The Principles and Practice of Rectal Surgery.* By WILLIAM B. GABRIEL, M.S.Lond., F.R.C.S. 2nd Edition. Price 28s.

*Chronic Streptococcal Infection as a Disease.* By J. D. HINDLEY-SMITH, M.A., M.R.C.S., L.R.C.P. Price 3s. 6d.

*Diseases of the Nose, Throat and Ear.* By J. DOUGLAS McLAGGAN, M.A., M.B., F.R.C.S. Eng. and Edin. Price 15s.

From OXFORD UNIVERSITY PRESS :

*Textbook of Gastroscopy.* By NORBERT HENNING. Translated by HAROLD W. RODGERS. Price 7s. 6d.

From P.E.P. (POLITICAL AND ECONOMIC PLANNING) :

*Report on the British Health Services.* Price 7s. 6d.

From SOCIÉTÉ DE PATHOLOGIE COMPARÉE, PARIS :

*Results followed up for twenty years after Grafting of the Thyroid in cases of Myxedematous Cretinism.* By SERGE VORONOFF. (Reprinted from *Bulletin de la Soc. de Path. Comparée,* No. 492.)

From JOHN WRIGHT & SONS LTD. :

*Physical Signs in Clinical Surgery.* By HAMILTON BAILEY, F.R.C.S. 6th Edition. Price 21s.

*British Journal of Surgery.* Vol. XXV. No. 98. (Oct., 1937.) Price 12s. 6d.

*First Aid to the Injured and Sick.* By WARWICK and TUNSTALL. Edited by F. C. NICHOLS, M.C. Price 2s. 6d.

### Local Medical Notes

His Majesty the King has conferred the honour of knighthood on Dr. William George Savage, M.D., D.P.H., late Medical Officer of Health for Somerset.

EXAMINATION RESULTS.

**University of Bristol.**—Students of the University have recently passed the following examinations :—

M.B., Ch.B.—*Second Examination* (*Section II.*). *Pass :* R. T. Davies.

M.B., Ch.B.—*Final Examination* (*Section I.*). *Pass :* W. H. G. Elliott, K. A. Hall.

M.B., Ch.B.—*Final Examination* (*Section II.*). *Pass :* R. L. J. Derham, H. D. T. Gawn, M. E. M. Herford, F. J. W. Hooper, G. L. Page (with Distinction in Public Health), Anna H. Silberstein. *In Group II.* (*completing Examination*) *:* P. N. Heron, P. K. Jenkins.

B.D.S.—*Second Examination. Pass : In Dental Anatomy* (*completing Examination*) *:* F. C. Baker, D. C. Bodenham.

B.D.S.—*Third Examination. Pass : In Dental Mechanics* (*completing Examination*) *:* A. A. Grant (with Distinction) G. R. Styles. *In Dental Materia Medica* (*completing Examination*) *:* G. H. Bell, J. E. H. Bell, J. F. Blenkinsop (with Distinction).

L.D.S.—*Final Examination. Pass :* H. B. Harding, J. C. F. Rolls.

L.D.S.—*Second Examination. Pass : In Anatomy and Physiology* (*completing Examination*) *:* L. B. Howell, P. Le Masurier, O. H. Minton. *In Dental Anatomy* (*completing Examination*) *:* J. C. Jowett, R. B. Stiles. *In Anatomy and Physiology only :* B. N. Neilson.

L.D.S.—*Third Examination. Pass : In Dental Mechanics* (*completing Examination*) *:* F. W. Clift, K. A. Gordon-Ralph, G. H. Hudson, E. L. Manton, Mary E. Thomas, May A. Trump, L. N. Weale, H. D. Williams, E. S. Wolfson. *In Dental Mechanics only :* J. W. S. Jenkins.

**University of Oxford.**—M.A. (by incorporation)—G. C. Williams, M.A. (Cantab.), M.R.C.S., L.R.C.P., D.P.H. (Brist.).

**Conjoint Board.** — L.R.C.P. M.R.C.S. — *Pass : In Physiology :* B. C. E. Aldous. *In Pharmacology and Materia Medica :* E. A. Griffiths, E. B. Hughes, J. I. Williams. *In Medicine :* R. L. J. S. Derham,* F. D. Mowat,* K. C. P. Smith,* Mabel W. N. Tribe.* *In Midwifery :* J. H. Bulleid, Margaret E. M. Slater. *In Pathology :* Mabel L. Glenny. *In Surgery :* K. G. Bergin.*

* Qualified.

### Index

Adams, A. W.—Spinal anæsthesia, 143.
Altitude, Therapeutic value of.—B. Hudson, 35.
Amalgamation of B.R.I. and B.G.H., 78.
Anæsthesia, Spinal.—A. W. Adams, 143.
Appointments :
F. G. Bergin, G. B. Bush and S. B. Adams.—B.R.I. and B.G.H. Radiological Department, 189.
A. W. Falconer.—Vice-Chancellor, Cape Town University, 238.
A. G. Palin.—Assistant Surgeon, Bristol Eye Hospital, 97.
Arrowsmith-Brown, J. A. — Obituary, 183.

Barbour, R. F.—Child guidance, 261.
Beddoes, Dr., and the Physical Fitness Campaign, 232.
Berry, R. J. A.—The Burden Mental Research Trust, 201.
Bodman, F.—Rôle of central nervous system in disease, 41.
Book Reviews, 64, 171, 227, 301.
Bristol Medical Dramatic Club, 98.
Bristol Royal Infirmary.—E. Watson-Williams, 167.
Bristol Royal Infirmary Bicentenary, 80, 190, 287.
Bristol Royal Infirmary and Bristol General Hospital Amalgamation, 78.
Elections to United Honorary Staffs, 182.
Burden Mental Research Trust.—R. J. A. Berry, 201.

Cardiac ischæmia.—L. O'Shaughnessy, 109.
Carey Coombs Memorial Lecture.—L. O'Shaughnessy, 109.
Casualty Clearing Station.—R. C. Clarke, 1.
Central Health Clinic, 231.
Central nervous system in disease.—F. Bodman, 41.
Child guidance.—R. F. Barbour, 261.
Clarke, R. C.—Evolution of a casualty clearing station, 1.
Colston Medical Research Fellowship, 82.

Drew-Smythe, H. J. — Prontosil in obstetrics, 217.

Examination Results, 96, 188, 237, 323.

Galenicals, 320.
Green-Armytage, V. B.—The debt of Western medicine to the East, 239.

Ham Green Hospital extension.—W. W. Jameson, 221.
Hudson, B.—Therapeutic value of altitude, 35.

Jameson, W. W.—Ham Green Hospital extensions, 221.

Library, 92, 185, 234, 321.

Macloghlin Scholarship.—D. S. Short, 190.

National Association for the Prevention of Tuberculosis. — Conference in Bristol, 181.
National Health Campaign, 313.
Nervous system in disease.—F. Bodman, 41.
Nixon, J. A.—Nutrition and tuberculosis, 191.
Nutrition and tuberculosis.—J. A. Nixon, 191.

Obituaries :—
J. A. Arrowsmith-Brown, 183.
George Parker, 82.
Arthur Walbank, 90.

Obstetrics, Prontosil in.—H. J. Drew-Smythe, 217.
O'Shaughnessy, L.—Cardiac ischæmia, 109.

Parker, George.—Obituary, 82.
Physical Fitness Campaign, Dr. Beddoes and.—232.
Poynton, F. J.—Research in medicine, 127.
Presidential Address.—R. C. Clarke, 1.
Prontosil in obstetrics.—H. J. Drew-Smythe, 217.
Prostate, Enlarged.—C. F. Walters, 21.
Publications Received, 94, 187, 236, 323.

Royal College of Surgeons of England—Macloghlin Scholarship.—D. S. Short, 190.

Spinal anæsthesia.—A. W. Adams, 143.
Sulphanilamide in treatment. — E. Watson-Williams, 209.

Territorial Medical Units in Bristol, 316.
Throat, A lump in the.—E. Watson-Williams, 279.
Tuberculosis Conference in Bristol, 180.
Tuberculosis, Nutrition and.—J. A. Nixon, 191.
Twenty-sixth Long Fox Memorial Lecture, 239.

U.S.S.R., Recent experimental work in.—F. Bodman, 41.

Walbank, Arthur.—Obituary, 90.
Walters, C. F.—Enlarged prostate, 21.
Watson-Williams, E.—How the B.R.I. has watched the world change, 167.
A lump in the throat, 279.
Sulphanilamide in treatment, 209.

